# Unclogged pores: designer channels for protein translocation

**DOI:** 10.1038/s42003-023-05723-z

**Published:** 2024-01-09

**Authors:** Jelle van der Hilst

**Affiliations:** Department of Biological Engineering, Massachusetts Institute of Technology, Cambridge, MA USA

## Abstract

Nanopores have the potential to revolutionize the field of protein sequencing, but due to the biochemical complexity of polypeptide sequences, they have remained mostly theoretical. In recent work, Sauciuc et al. engineer the protein nanopore CytK to produce an electroosmotic force capable of translocating unfolded polypeptides regardless of their charge distributions, an important step toward single-file protein nanopore sequencing.

Recent advances in DNA sequencing technology have revolutionized modern genetic research, including full sequencing of the human genome, exploration of the rich environmental metagenome, and tailored genetic medicine. However, protein sequencing technologies lag far behind their nucleic acid counterparts, limiting efforts to map harmful mutants, low-abundance proteins, and comprehensive proteomes of cells (including post-translational modification status not predictable from genetic sequence alone). Due to their complicated 3D folding, far greater molecular complexity, and irregular charge distributions, proteins present a challenge to sequence. Current approaches such as Edman degradation and mass spectrometry are low throughput and expensive. New work from Sauciuc et al.^1^ cleverly adapts nanopore technology to scan single-file unfolded polypeptides, a step toward linearized protein sequencing.

Molecular sequencing nanopores can be synthetic or biological; for example, many studies use *β*-barrel proteins set in membranes. To drive a molecule through, a voltage is applied across the pore; this electrophoretic force (EPF) drives the translocation and detection of the molecule. The EPF alone, however, does not provide enough force to translocate polypeptides due to their inconsistent or weak charges. The authors sought to leverage another force, electroosmotic force (EOF), which generates flow through the nanopore due to the net charge of the pore. One challenge is that the CytK *β*-barrel pore used in other protein translocation studies does not have a net internal charge, which prevents EOF generation. To overcome this issue, the authors design and test a series of charged CytK mutants. By decorating the interior of the channel with negatively charged residues, they generate an EOF sufficiently strong to force a polypeptide through the pore even when the EPF runs the opposite direction (Fig. 1a). The most successful pore mutant has three evenly spaced rings of negative charges and generates an EOF capable of translocating both highly positively and highly negatively charged unfolded proteins. The authors also attempt to construct a positively charged pore mutant decorated with lysines; however, this version of the pore failed to translocate any of the test proteins. Finally, long and structured proteins such as the 421-residue maltose binding protein, unfolded with urea or guanidine hydrochloride, are passed through the engineered pore with similar efficiency as natively unfolded proteins; this result confirms that this pore has a versatile ability to translocate any polypeptide sequence once unfolded.

**Fig. 1:**
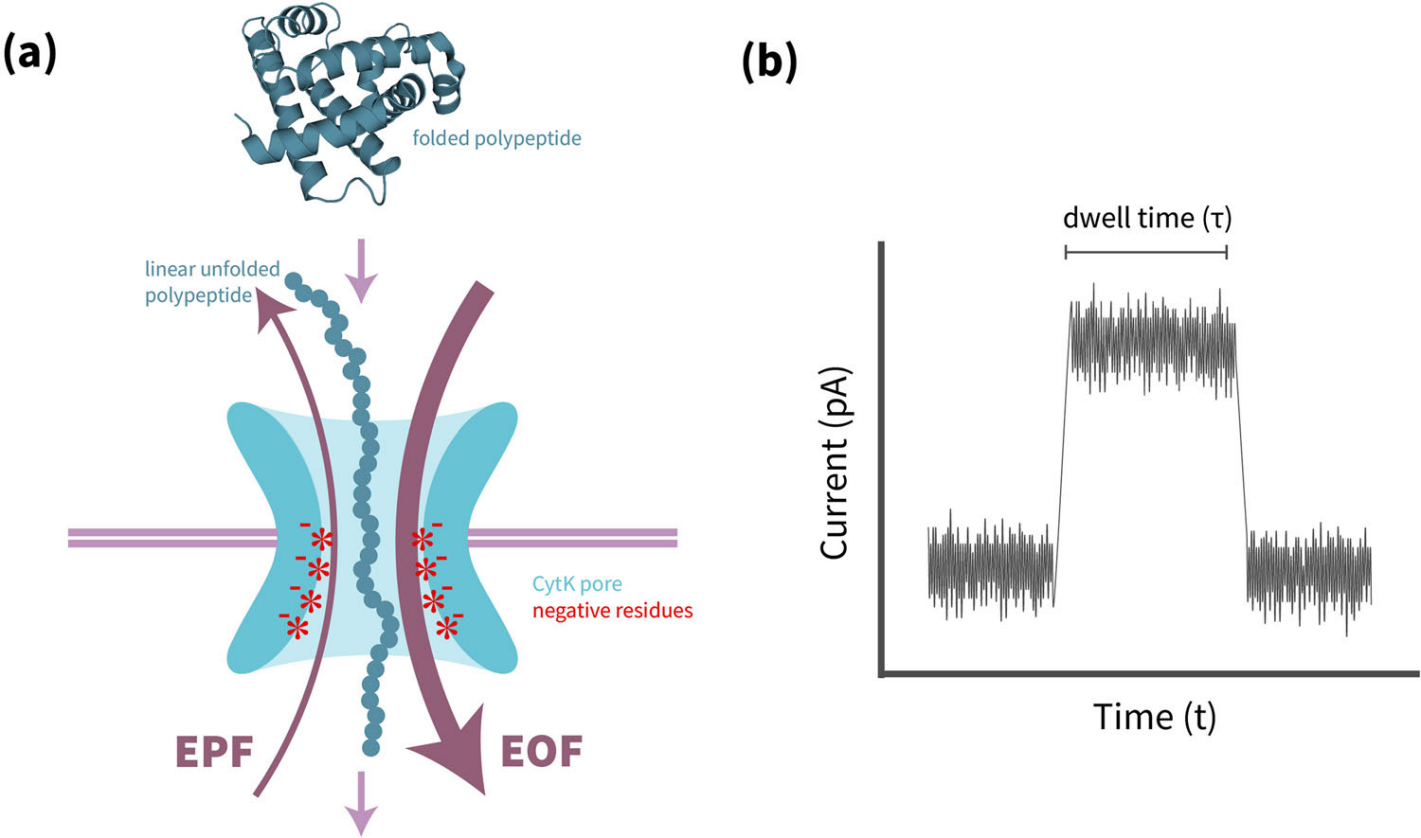
The modified CytK pore drives translocation of unfolded polypeptides; this translocation can be measured as a change in current. **a** The negative charges of the 2E-4D modified CytK (cyan) create a strong EOF capable of translocating any linear, unfolded polypeptide (dark blue), even when the EPF has an opposite direction. Myoglobin is shown here as an example (PDB ID 1MBN) shown as an example, rendered in Pymol. **b** The passage of a linear polypeptide can be measured by changes in the current across the pore; an example trace is shown here with the polypeptide translocating during the trace peak (*τ*).

Promisingly, the authors are able to detect subtle changes in current along the length of the transduced polypeptide, which they suggest could correspond to individual residues or leftover structural motifs from imperfect unfolding (Fig. 1b). Further research is needed to characterize this effect and decode these impedance patterns into distinguishable signals for each of the 20 canonical amino acids and their post-translational modifications. While this technique is still far from achieving single-amino-acid resolution sequencing of linearized polypeptide chains, the engineered pores shown in this study set the stage for nanopore protein sequencing, with the potential to replace dated and expensive techniques.
